# Factors leading to lapses in professional behaviour of Gynae residents in Pakistan: a study reflecting through the lenses of patients and family, consultants and residents

**DOI:** 10.1186/s12909-024-05509-9

**Published:** 2024-06-03

**Authors:** Humera Noreen, Rahila Yasmeen, Shabana Ali Mohammad

**Affiliations:** 1 Department of Obstetrics &Gynaecology, Rawalpindi Medical University, Rawalpindi, Pakistan; 2 Department of Medical Education, Riphah International University, Islamabad, Pakistan

**Keywords:** Lapses in professional behaviour, Professionalism, Gynae residents, Consultants, Factors and reasons, Patients and family

## Abstract

**Introduction:**

Professional behaviour is the first manifestation of professionalism. In teaching hospitals, the residents can be considered vulnerable to lapses in professional behaviour when they fail to meet the set standards of professionalism. Residents of some specialties are more at risk of lapses in professional behaviour due to the demanding nature of work. Research focusing on the behaviour of residents in the field of Gynae and the underlying factors contributing to such behaviour is notably lacking in the literature. Additionally, there is a gap in understanding the perspectives of patients from Pakistan on this matter, as it remains unexplored thus far, which constitutes the central focus of this study.

An increase in complaints lodged against Gynae resident’s professional behaviour in Pakistan Citizen Portal (PCP) was observed. Therefore, an exploratory qualitative study was conducted to investigate the factors and rationales contributing to the lapses in resident’s professional behaviour.

The study collected the viewpoints of three stakeholder groups: patients and their families, consultants and residents. The study was conducted in three phases. First, the document analysis of written complaints was conducted, followed by face-to-face interviews (11 per group) conducted by trained researchers from an independent 3rd party. Finally, the interview data was transcribed, coded and analysed.

In total 15 themes were identified from the interviews with 3 stakeholders, which were then categorized and resulted in 6 overlapping themes. The most prevalent lapse reported by all 3 stakeholders was poor verbal behaviour of residents.

**Conclusion:**

The highly ranked factors contributing to triggering the situation were associated with *workplace challenges, well-being of residents, limited resources, patients and family characteristics, patients’ expectations, lack of administrative and paramedic support, cultural factors and challenges specific to Gynae specialty*.

Another intriguing and emerging theme was related to the *characteristics of patients and attendants* which helped in understanding the causes and implications of conflicting environments. The value of competency also emphasized that can be accomplished by training and mentoring systems. The thorough examination of these factors by key stakeholders aided in accurately analysing the issue, its causes, and possible solutions. The study's findings will assist higher authorities in implementing corrective actions and offering evidence-based guidance to policymakers to improve healthcare system.

## Introduction

Understanding human behaviour is very important to comprehend how people see, interpret and adapt to various environments and to have an insight about the reasons people change their behaviour [1]. The same principle applies to professional behaviour (PB) in workplace environments including healthcare. The professional behaviour in a healthcare system could be defined as placing the best interests of patients at the center of everything you do [2].

Lapses in professional behaviour lack a unified definition, one could say that any behaviour that impairs the ability of the medical team to achieve desired outcomes is considered as lapse in PB or when someone is not following the standards expected from a person in their profession and behaving against them. Lapses could also be taken as a behaviour characterized by actions that a reasonable person would view as humiliating, rude, disrespectful, abusive language, demeaning, and bullying [3]. Lapses in professional conduct not only impact patient-doctor relationships, patient safety, and the quality of care but also the doctor's career [4].

According to Accreditation Council for Graduate Medical Education (ACGME) guidelines, residents must acquire professional behaviour as a core competency. The expected domains of professional behaviour projected by the residents include empathy, honesty, and respect for others [5]*.* Residents are susceptible to engaging in a variety of lapses in professional behaviour throughout their residency, which is a crucial time for doctors to build their ethical norms. It is essential to have a complete awareness of the relevant causes or events in order to prevent resident misbehaviour [6].

There is a scarcity of literature specifically examining the assessment of trainees' reasons and varieties of professional conduct lapses [7]. In the existing body of literature, the behavioural deficiencies observed among residents encompass various misconduct aspects, including disruptive practices involving patient care neglect, absence of empathy, and disrespect toward patients, as well as verbal or nonverbal misuse of authority and unwelcoming demeanor [8–10].

However, it's been acknowledged that addressing lapses in professional behaviour poses one of the most difficult challenges for medical educators. Understanding the underlying reasons behind such lapses is intricate, with the *context* in which the behaviour manifests playing a pivotal role [7].

Research indicates a connection between unprofessional conduct during undergraduate and postgraduate training and similar behaviour in later practice [11]. A conceptual framework has been proposed for evaluating and addressing lapses in PB [3]. To identify the root causes of PB lapses, it is crucial to determine whether residents understand professional expectations and are willing to adhere to them, or if there are barriers or distractions hindering their ability to exhibit professional behaviour [12]. The problem of lapses in PB is not specific to any specialty and appears to be occurring in various demographic groups/countries [13–15].

In teaching hospitals tasked with training new physicians, the diverse background of patients and their attendants often leads to an overwhelming environment. This diversity breeds a behavioural gap, primarily driven by the varying interests of individuals involved. Patients and their attendants bring different levels of understanding, influenced by educational exposure, personal values, and background, which can disrupt interactions and impact patient-doctor relationships [16].

Furthermore, residents face additional pressures that contribute to their susceptibility to unprofessional behaviour. The urgency to quickly acquire clinical knowledge during their residency often supersedes their focus on professional conduct, leading to a lack of understanding in this regard [17, 18]. Additionally, residents may feel compelled to remain silent about professional challenges they encounter during their training to uphold the hospital's public image [14]. Therefore, it becomes a difficult task for medical tutors to unfold the truth and deal with it in the best interest of doctors and patients.

Several studies reported by developed countries have explored various facets of professionalism in alignment with their respective healthcare systems and policies [13, 15, 18]. However, it is crucial to recognise that the findings of these studies may not be directly applicable to developing countries without appropriate contextual adaptation.

As a developing country, Pakistan presents a unique set of factors that differ from those observed in developed nations, including government policies, patient awareness, medical practices, doctor workload, and cultural influences. Furthermore, there is a scarcity of data regarding patient perceptions of PB lapses, the underlying reasons for inappropriate resident behaviour, and the specific contextual factors within the local environment that influence resident conduct.

Research indicates that certain medical specialties, such as OB-GYN and Surgery, face increased risks of specific unprofessional behaviours due to their distinct stressors [14]. Therefore, it is suggested to conduct context-specific assessments to identify and mitigate these stressors effectively [19]. A narrative study conducted in King Edward, Lahore Pakistan aimed to explore conflicts in Obs and Gynae, revealing organizational, interpersonal, and individual conflicts [20]. Further investigation is required to understand the underlying factors and triggers of these conflicts. While various factors influencing professionalism in junior medical professionals have been identified, there is a notable gap in research focusing specifically on Gynae residents. A recent study has reported high rates of unprofessional behaviour among dismissed General Practice residents (90%), with a significant proportion displaying disrespect towards patients or staff (27%) [21]. Unprofessional behaviour among doctors is regulated by disciplinary bodies of respective country which have extensive effects on doctors health and career. A higher incidence of disciplinary actions noted in Obstetrics and Gynaecology [22]. Majeed et al. reported in the narrative review that issues related to poor ethical behaviour by trainees are usually presented as patient’s complaints. Disrespectful behaviour (worrisome communication, reluctant to talk, behavioural change affecting patients and family) was one of the most reported unprofessional behaviour in the narrative review [23].

## Context

The study's context revolves around a non-profit public sector medical university situated in Rawalpindi (population range of 1,000,000–5,000,000 inhabitants), Punjab, recognized by the Higher Education Commission of Pakistan [24]. The medical university operates as a small-sized coeducational university (uniRank enrollment range: 3,000–3,999 students) with significant Obstetrics and Gynae patient workload across three allied hospitals [25]. Due to the overwhelming patient load and resource constraints, unresolved complaints and conflicts have arisen. To address this, Prime Minister's Performance Delivery Unit (PMDU) [26] initiated a national complaints and grievance redressal mechanism called Pakistan Citizen Portal (PCP) [27]. (https://citizenportal.gov.pk/), aiming to provide citizens with a streamlined platform for resolving complaints efficiently. A substantial number of complaints were filed against Obs and Gynae residents, prompting the need to investigate the underlying reasons for such grievances. Existing literature highlights the importance of professional behaviour in residents, yet studies specifically targeting Gynae residents' conduct and the reasons behind lapses are lacking. Moreover, the perceptions of Pakistani patients, the primary stakeholders affected, remain unexplored, along with the comparative analysis of opinions among the three main stakeholders (patients, residents and consultants) to unveil the true circumstances surrounding the issue.

### Study aims and research questions/objectives

- ⊳ Research questions
  1. What are the lapses in professional behaviour of Gynae residents?
  2. What are the factors and reasons leading to the lapses in professional behaviour of Gynae residents from the perspectives of patients and family, Gynae consultants and residents in Pakistani context?
- ⊳ Objectives
  1. To determine the frequency of identified lapses in professional behaviour among the Gynae residents by analysing PCP complaints
  2. To explore the factors and reasons leading to the lapses in professional behaviour of Gynae residents from the perspectives of patients & families, Gynae consultants and residents.

A comprehensive analysis was done which aided in accurately diagnosing the problem, identifying its root causes, and devising effective remedies. The findings of this study are considered key enablers for the higher authorities to implement corrective measures and offer evidence-based guidance to policymakers, thereby enhancing the overall healthcare system.

## Methods

### Study design

**Fig. 1:**
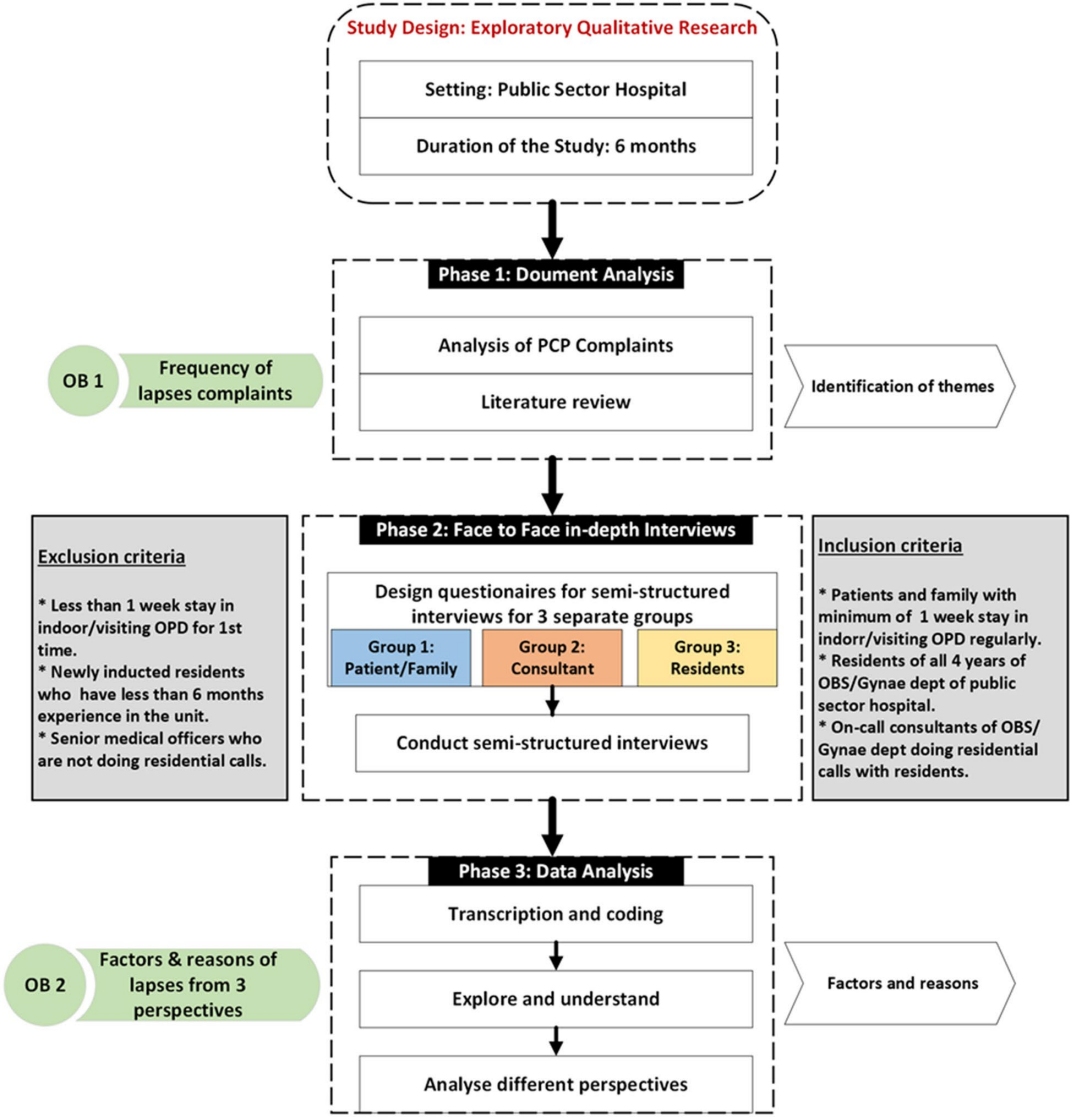
The Conceptual framework of our study. Note: OB1 (Objective 1), OB2 (Objective 2)

The conceptual framework of this study is illustrated in (Fig. 1) and represents an exploratory qualitative approach, commonly employed in education and social sciences research [28]. This qualitative study spanned a duration of 6 months, commencing from Dec 2021 till June 2022, and was conducted within two Obs and Gynae departments within the public healthcare sector in Rawalpindi, Pakistan.

Two research methodologies were utilized: document analysis of PCP complaints to identify and confirm the frequency of lapses in professional behaviour among Gynae residents,

**(Phase 1)**, and in-depth interviews designed on the basis of findings of the document analysis **(Phase 2)**. The in-depth interviews, conducted with the written consent of the participants, aimed to delve deeper into the perspectives of various stakeholders regarding lapses in professional behaviour and their underlying causes, providing contextual meaning [29]. Document analysis inform the design of the interview schedule. The study was approved by the institutional review committee of Islamic International Medical College (Ref No Riphah/IIMC/IRC/22/2003; approval date: 27/01/2022).

### Participants

The study population consisted of three groups.

- Patients and family
- Consultants
- Residents

The recruitment of study participants employed purposive sampling, aiming to select individuals or groups highly knowledgeable or experienced in the phenomenon under investigation. Criteria included availability, willingness to participate, and the ability to articulate experiences and opinions effectively [30]. Participants were chosen from two public sector hospitals in Rawalpindi, Pakistan, comprising stable patients and family members with a minimum one-week hospital stay, residents with over six months of training in the Obs & Gynae department, and consultants engaged in residential calls alongside residents. To ensure the comfort and security of female patients during interviews, both the patient and her husband were included in the first group. Exclusions included patients with less than one-week hospital stay, those deemed unstable or unwilling to engage, newly inducted residents with less than six months of training, and senior medical officers not involved in residential calls. A total of 33 semi-structured interviews were conducted, 11 per group, to explore factors contributing to lapses in professional behaviour. Saturation was achieved by the 10th interview, with one additional interview conducted for confirmation.

### Data collection

The data collection was done in a structured manner in two phases.

### Phase 1: (The complaints data and document analysis)

In phase 1, complaints uploaded to the PCP portal were examined to address the first objective of this study. Access was granted to a nominated Deputy Medical Superintendent (DMS), who regularly logged in to the Citizen Portal website to review complaints. Specific complaints against residents involve prompt notification of the focal persons in the concerned department, who immediately addressed the issue by investigating the resident and their consultant. Following fact-gathering, complainants were invited for scheduled meetings to evaluate allegations and attempt resolution; unresolved complaints were escalated to higher authorities.

This study's initial step involved confirming the number of complaints against Gynae residents regarding their behaviour. Formal permission was obtained to access all complaints, facilitating the review to determine complaint frequency and comprehend complainants' messages for administrative authorities. Complaint phrases were consolidated into themes, supplemented by additional themes identified in the literature, laying the groundwork for designing a semi-structured interview questionnaire. Complaints lodged by patients and families were documented in Table 1, highlighting lapses in professional behaviour as a primary concern.

**Table 1:** Pakistan citizen portal complaints (PCP)

S.NO	PCP Complaints (*N* = 38)	Frequency
1	Poor verbal/Rude behaviour of resident	10
2	Lack of facilities	09
3	Lack of care/attention by Doctor	03
4	Poor verbal/Inappropriate behaviour by nursing staff in labor room	03
5	Non availability of the senior consultant	02
6	Long working hours of residents	02
7	Long waiting hours of patients	02
8	Huge burden of the patients	01
9	Lack of appropriate number of the doctors	02
11	Doctors get salaries from taxes paid by patients but don’t provide proper care	01
12	Bribery by junior staff (Aya)	01
13	Refusal to attend the patient in emergency by Gynae department	01
14	Poor verbal communication by DMS	01

### Phase 2: (Interview data)

#### Interview structure

In the second phase of data collection, in-depth interviews were conducted for two primary objectives. Firstly, to reconfirm the nature of complaints against Gynae residents, and secondly, to delve deeper into the underlying issues from three distinct perspectives.

Semi-structured one-to-one interviews were chosen as the research instrument due to the sensitive nature of the topic. Three separate interview guides were developed for patients and their families, as well as residents and consultants, following the structure of the AMEE guide 87 [31].

Special attention was paid to ensuring that the language used in the interview guides was clear and understandable. The guide for patients was prepared in Urdu, while those for consultants and residents were in English. All interviews were conducted in Urdu to accommodate the participants' comfort in expressing their views and were later translated as needed.

#### Expert validation and pilot testing

The questionnaire used in the interview guide underwent expert validation by four Medical Educationists, who possessed a minimum of 5 years of experience. This validation aimed to assess the clarity and relevance of the questionnaire items in relation to the construct being measured. Feedback provided by the experts led to modifications in some items and adjustments in their sequence.

Following this, *pilot testing* was conducted, involving one participant from each stakeholder group. The results of the pilot interviews prompted further refinements in the interview guides and probes.

The interview guide consisted of **two parts**: the first part focused on gathering demographic information, while the second part addressed the study objectives. The demographic section collected details such as age, education, profession, duration of marriage, and family structure. Patients and their families were asked seven questions, while residents and consultants were presented with ten questions, aiming to capture information relevant to the research objectives.

Through this investigation, not only were the specific instances of lapses in professional behaviour among residents examined, but also the broader spectrum of issues that patients raised in their complaints.

#### Bias control

Considering the sensitivity of the study topic and the senior position of the researcher within the organizational hierarchy, *third-party researchers* were engaged to conduct the interviews. Each of the three stakeholder groups was interviewed separately. These face-to-face interviews were conducted following rigorous protocols to ensure accuracy and reliability. To mitigate **social desirability bias** [32], which could skew responses, the interviews were conducted by a separate team of psychologists who were not affiliated with the organization being studied. This team comprised three members, with each member assigned to interview one of the stakeholder groups, allowing them to develop a better rapport with respondents over time.

Logistics for the interviews were managed by a liaison person from the hospital, ensuring smooth coordination. Participants were provided with information sheets detailing the study aims, interview process, and assurances of data confidentiality and anonymity. The interviews were conducted in a designated space within the hospital premises and began after obtaining written informed consent from each participant. Audio recordings of the interviews were made, with an average duration of 45 min per session.

Challenges encountered during the interviews were addressed through daily online meetings, allowing the interviewers to discuss and overcome any issues faced. Patients and their families initially exhibited reluctance to participate, but counseling and assurances of confidentiality helped alleviate their concerns. For residents, anonymity was assured by assigning specific IDs and group codes to the transcribed interviews.

Each interview was transcribed verbatim and provided to the main researcher for analysis. Similar sets of questions were posed to all three stakeholder groups, aiming to elicit their perspectives on the factors influencing lapses in professional behaviour.

### Phase 3: (Data analysis—theme extractions)

**Fig. 2:**
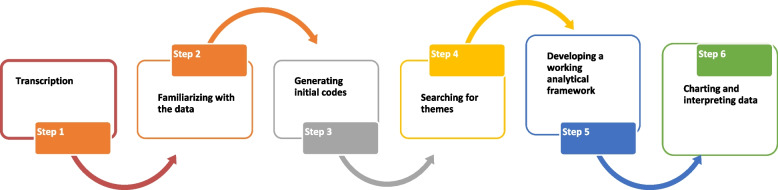
Steps of framework method of thematic analysis

In phase 3, the framework method of thematic analysis was employed, known for its structured approach facilitating systematic comparison of data across cases [33]. This method involves several steps, as depicted in Fig. 2.

Firstly, the interview data was transcribed (Step 1), followed by familiarization with the transcriptions (Step 2). Initial codes were then generated to capture key concepts and ideas (Step 3), which were subsequently organized into themes, which aligned with the themes identified in the literature (Step 4). Additionally, new themes emerged during this process, enriching the analysis.

A working analytical framework was developed based on these themes (Step 5), and the final step involved interpreting the data within this framework to derive meaningful insights.

#### Quality and transparency

The study's expenses, which involved hiring third-party researchers, were solely supported by personal finances, without any contribution from public funds. Each researcher dedicated six days to conduct interviews with a total of 33 participants.

To ensure the quality of this study, several *triangulation strategies* were employed [34]. *Credibility* was enhanced through data triangulation by gathering information from three different stakeholder groups. Investigator triangulation involved the participation of multiple researchers, while method triangulation utilized both document analysis and semi-structured interviews.

To establish *trustworthiness*, transcripts were reviewed by additional researchers, and consultations were held with peers, study supervisors, and co-supervisors throughout the data processing stages. Thick descriptions were provided to elucidate behaviours, experiences, and contextual factors, increasing the study's *transferability* while acknowledging potential contextual differences.

The study's methodology was transparent, with each research step meticulously described from inception to reporting, ensuring *dependability*. Detailed records were maintained throughout the study, contributing to the transparency and reliability of the findings.

#### Team reflexivity

Semi-structured interview guide prepared by HN, main researcher and Gynaecologist by profession. Reviewed by RY, who is Dean of medical education department. Interviews were conducted and transcribed by US, AT and BM who are psychologists. Thematic analysis done by HN (Gynaecologist) and AG (who is also psychologist). Results were reviewed by LE (medical educationist) and FS (PhD and trained in qualitative research). The reflexivity was ensured by critical self-reflection about oneself as researcher and making the position of the researcher and the participants clear. The methodology explained in detail.

## Results

The results are described in **2 parts**. The **1st part** is the **complaint data** from document analysis of complaints on Pakistan Citizen Portal (PCP). The **2nd part** explains the findings from the **interview data** sets.

### Part 1-Complaint data

#### Participant characteristics for document analysis

All the complaints are anonymised and can't be tracked back—no demographic data available which lead us to re-verification of these complaints.

The literature shows that complaints provide an opportunity to understand the problems and the ways these can be resolved [27]. In PCP document, the range of complaints were diverse, 26% (*n* = 10/38) were against resident’s professional behaviour which is the core investigation of this study. The rest were related to lack of facilities, infrastructure, and lack of facilities for attendants and huge workload. (Table 1).

### Part 2- Interview data

#### Participants characteristics for the interviews

The following Table 2 shows the demographic of all 3 stakeholders.

**Table 2:** Participant’s characteristics for interviews

Characteristics	**Mean (SD)**	**Percentage**
**Demographics of Patients ** ***N*** ** = 11)**		
Age (years)	30–36 (30.9 ± 5.41)	
Marriage (in years)	10 ± 6.2	
Education (in years)	9.6 ± 2.34	
Occupation (Housewife)	*n* = 11	100%
Gender (female)	*n* = 11	100%
Family system		
Nuclear system	*n* = 5	45%
Joint system	*n* = 6	54%
**Demographics of consultant (*N*** ** = 11)**		
Age (years)	37–44	
Married	*n* = 11	100%
Education (FCPS)	*n* = 11	100%
Clinical experience (> 10 years)	*n* = 11	100%
Family system		
Nuclear system	*n* = 2	18%
Joint system	*n* = 9	82%
**Demographics of Residents (*N*** ** = 11)**		
Gender		
Females	*n* = 10	91%
Male	*n* = 1	10%
Year of training		
3rd year residents	*n* = 8	73%
2nd year residents	*n* = 3	27%
Marital status		
unmarried	*n* = 5	45%
married	*n* = 6	54%
family system		
nuclear	*n* = 7	64%
joint	*n* = 4	36%

#### Reinforcing evaluation of the identified themes in PCP Document

The first question of interview was asked to re confirm the identified themes in PCP document. This part of the investigation provides a wider perspective of all three stakeholders related to the frequently observed/faced complaints (Table 3). Interestingly, the poor verbal communication and behaviour has been listed at the top with the highest value which reinforces the importance of this study. Other issues highlighted were lack of attention and insufficient communication by doctor and the complaints related to labour room. Few of the complaints were against paramedical and lower staff (Table 4).

**Table 3:** Lapses of professional behaviour identified by different stakeholders

Lapses in professional behaviour identified by different stakeholders					
Patient’s Perspective	Frequency	Consultant’s perspective	Frequency	Resident’s Perspective	Frequency
Poor verbal behaviour of resident	08	Poor verbal communication	08	Poor verbal communication	09
Strict/harsh behaviour of paramedical staff	06	Unprofessional behaviour of residents	06	Doctors inappropriate behaviour	06
Insufficient attention by the residents	04	Unsatisfactory counseling skills	05	Poor non-verbal communication	06
Disrespectful behaviour in labor room	04	Ignorant behaviour of residents	05	Low level of satisfaction among patients	05
Doctor Advice Expensive tests	03	Allocation of insufficient time	05	Unsatisfactory counseling skills	05
Insufficient info to the patients	03	Scolding by the resident	04	Poor time allocation per patient	04
Commendation	03	Poor Nonverbal communication	03	Delay in treatment	04
Negligence	02	Poor communication skills	03	Complaints related to Labor Room	04
Behaviour difference based on social class	01	Complains related to labor room	03	Poor Communication skills of residents	03
		Complaints regarding the guidance of treatment plan	01	Lack of empathy	03

**Table 4:** Other complaints by patients identified by different stakeholder

Other complaints by patients identified by different stakeholders					
**Patient’s Perspective**	**Frequency**	**Consultant’s perspective**	**Frequency**	**Resident’s Perspective**	**Frequency**
Prolonged Waiting time	07	Patients characteristics	07	Poor infrastructure	03
Strict/harsh behaviour of paramedical staff	06	Complaints about doctors’ triage protocol	03	Attendants complaints regarding the arrangement of blood and medicine	03
Lack of facilities for attendants	05	Low comprehension level of patients	03	Prioritization of the high risk patients	02
Lack of availability of Doctor during visiting hours	04	Extended waiting hours	03	Attendants’ un satisfaction with resident	02
Financial constrains because of prolonged hospital stay	03	Unrealistic expectations from residents	02	Complains related to paramedical staff	02
Lack of guidance by guard	01	Limited resources	02	Presence of male residents in labor room	01
Language barrier	01	Un cooperative /harsh nonverbal behaviour paramedical staff	03		
Restriction of male attendant in the ward	01	Forced arrangement of required medicine	01		
Blood arrangement	01				

Complaints other than lapses of professional behaviour includes prolonged waiting time, poor infrastructure and limited resources. (Table 4).

#### Findings

The interviews provided the researcher with rich insights into the factors and associated reasons for lapses in professional behaviour. Detailed findings are presented in the form of extracted themes and sub-themes for each stakeholder along with associated reasons.

### Grouped classification of themes

**Fig. 3:**
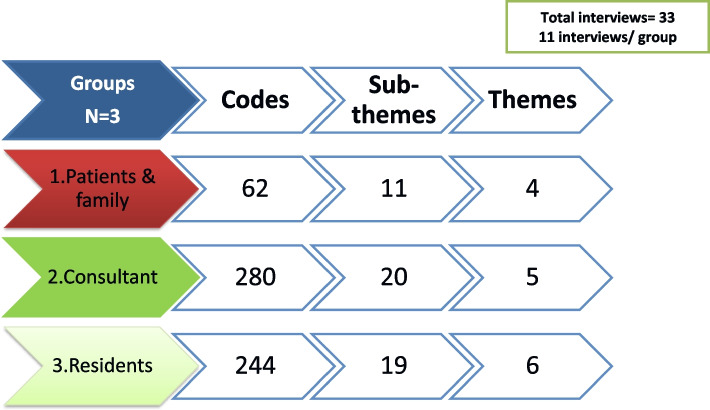
Total themes, subthemes and codes extracted on analysis of data from 3 stakeholders

The analysis was initiated by employing thematic analysis framework, as described in section of data analysis to examine data from three distinct stakeholder groups. Just to clarify, in this context, the term "themes" refers to the factors that each stakeholder perceived as potential contributors to professional lapses. Total; 15 themes extracted on analysis of data from 3 stakeholders (Fig. 3).

**Fig. 4:**
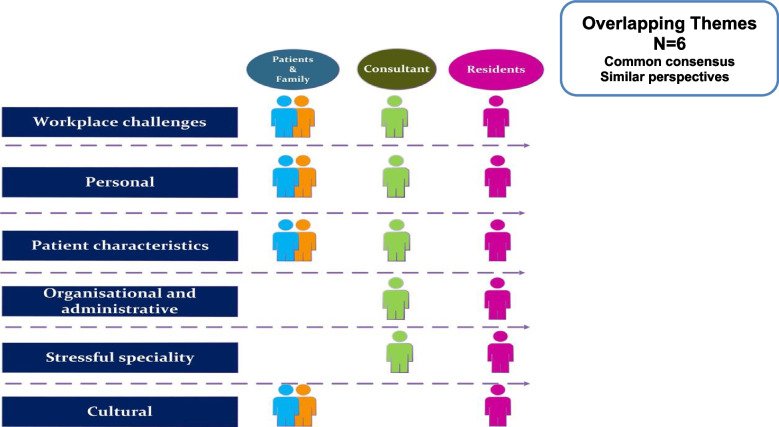
Six overlapping themes from 3 stakeholders

**Fig. 5:**
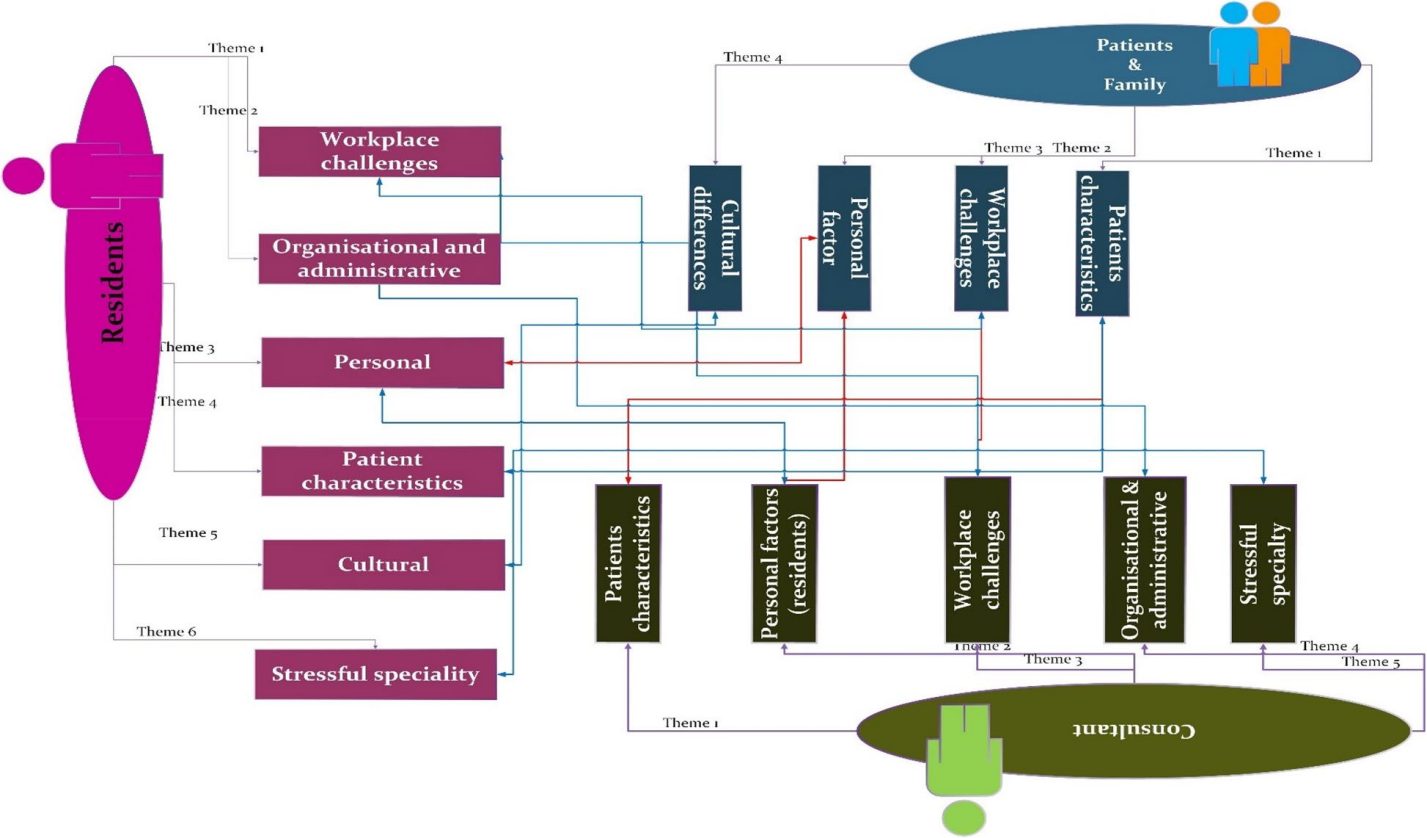
Mesh of overlapping themes (grouped classification of common themes)

The identified themes and sub-themes from the perspectives of the three stakeholders revealed some commonalities. There are a total of *six overlapping themes* (these are the themes with common consensus and similar perspective). These are discussed in detail later in this section and presented in (Fig. 4), indicating the stakeholders with whom these themes overlap. These overlapping themes help in shedding the light on the issue from 3 different angels. A graphical representation of the overlapping themes is depicted as a mesh in Fig. 5.

In this section, we delve into the six overlapping themes, exploring the analysis results by comparing the perspectives of the three stakeholders. This comprehensive approach provides insights into each theme from various angles, shedding light on the factors influencing lapses in professional behaviour among residents. Additionally, notable quotes are incorporated to offer a vivid and relatable depiction of these findings, enhancing understanding and authenticity.

#### Theme 1: Personal

**Fig. 6:**
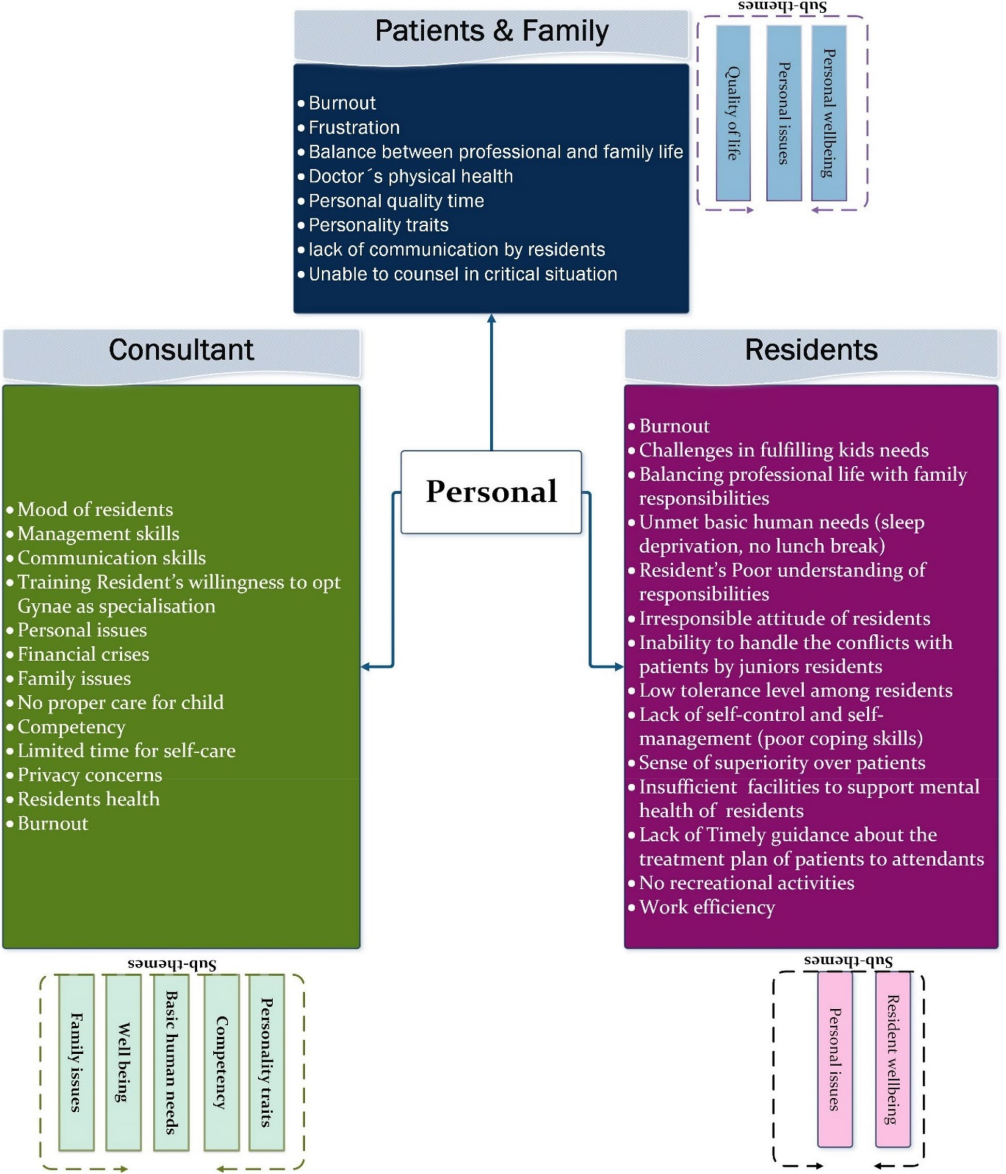
Presenting the reasons associated with the common theme ‘Personal’

The first overlapping theme, depicted in Fig. 6, illustrates the sub-themes and perspectives of stakeholders regarding this common theme. It showcases stakeholders' viewpoints on potential issues contributing to lapses, closely linked with their ‘personal needs and concerns’. These encompass family concerns, caring responsibilities, individual personality traits, basic human needs, quality of life considerations, competency levels, and overall well-being.

For majority residents, balancing caring responsibilities for young children without sufficient family support poses a significant challenge, adding to the stress of professional obligations. Additionally, residents experience frustration and disappointment when they are unable to meet their child's or their own health-related needs adequately.

[Resident] "Family support is very big factor. For example, there is a female doctor and she has children, so if there is a problem at home, it obviously affects the doctor."ID_03

The residents interviewed expressed concerns about their heavy workload, which has adverse effects on their emotional and physical well-being. This indicates the importance for hospital management and administration to devise strategies to address the specific needs of residents and provide them with adequate support for their unavoidable personal responsibilities.

[Resident] “We have mother residents who have to breastfeed their babies and sometime they did not even get time to pump milk for their babies” ID_01

[Resident] “As a Gynae resident we cannot spend proper time with our family. We have 30 to 32 hours so whatever time we have in our house we have to sleep at that time. ID_09

**Personal factors** such as childcare and household responsibilities have also been emphasised by both patients and consultants as potential triggers for behavioural issues.

[Patient & Attendant] “doc is also human she has to perform all her professional duties along with home responsibilities of taking care of kids and home.” ID_10

[Consultant] "Some of our doctors are very good, counsel the patient properly, talk comfortably and some doctors are in a bad mood in the morning, they may have their own problems that affect them, so they get tired."ID_1.

Many females in Gynae face challenges in securing support from their in-laws and family to manage their households. When in-laws fail to understand the demands of the profession or are not present in the same city, and hiring domestic help is difficult and costly, these stressors can significantly affect their performance and attitude towards their professional responsibilities.

[C] “Doctors mostly have their own tension going on. They may already be suffering from some tension that is affecting them”ID_3

**Well-being** serves as a vital metric in fostering a positive workplace environment. In the context of this study, we focus on the well-being of residents tasked with serving in the healthcare sector, particularly in the demanding field of Gynae. Recognized as a central aspect across various themes within this study, well-being emerges as a shared concern among all stakeholders, potentially contributing to lapses in professional behaviour.

[C] “if the resident is not feeling well that will also influence her behaviour with patients “ID_5”

The interview findings have outlined several influential factors impacting residents' well-being, with the most prominent being the long working hours, stringent duty schedules, and sacrifices made regarding basic human needs.

[R] There should be any stress relieving activities or vacations for us to spend time with our family. ID_07

[P] Residents are human and they are working a lot so they can get tired by working so much.” ID_09

[R] You cannot take off because it is a big issue and because of that you cannot have mental break from this entire situation. ID_0

There is a pressing need for institutional-level interventions to address various associated factors and support the well-being of residents. This will create a more conducive working environment that positively influences professional behaviour.

[R] “"it’s on the institutional level, patient load and tough routine. We are here for almost thirty six hour; we do not get short breaks for lunch and sleep. We are not machines we are humans, even then we try to give our 100%" ID_04.

**Competency** is pivotal in managing workload and meeting patients' needs. Notably, patients have never complained about treatment, reflecting positively on professional competency of residents. However, deficiencies in counseling skills, multitasking, and handling conflicts are observed. Consultants discern differences in attitude between senior and junior residents, highlighting the need for tailored training to equip residents for diverse situations.

[Consultant] “Experienced resident will stay cool and calm, and she knows that how to deal with things or how to answer about issues”ID_5

The management should implement a mentoring scheme to train junior members, offering them opportunities to shadow their seniors. Analysis of interview data strongly suggests that the interpersonal theme is closely linked with competency, the demands of the Gynae specialty, and administrative/management factors.

#### Theme 2: Workplace challenges

**Fig. 7:**
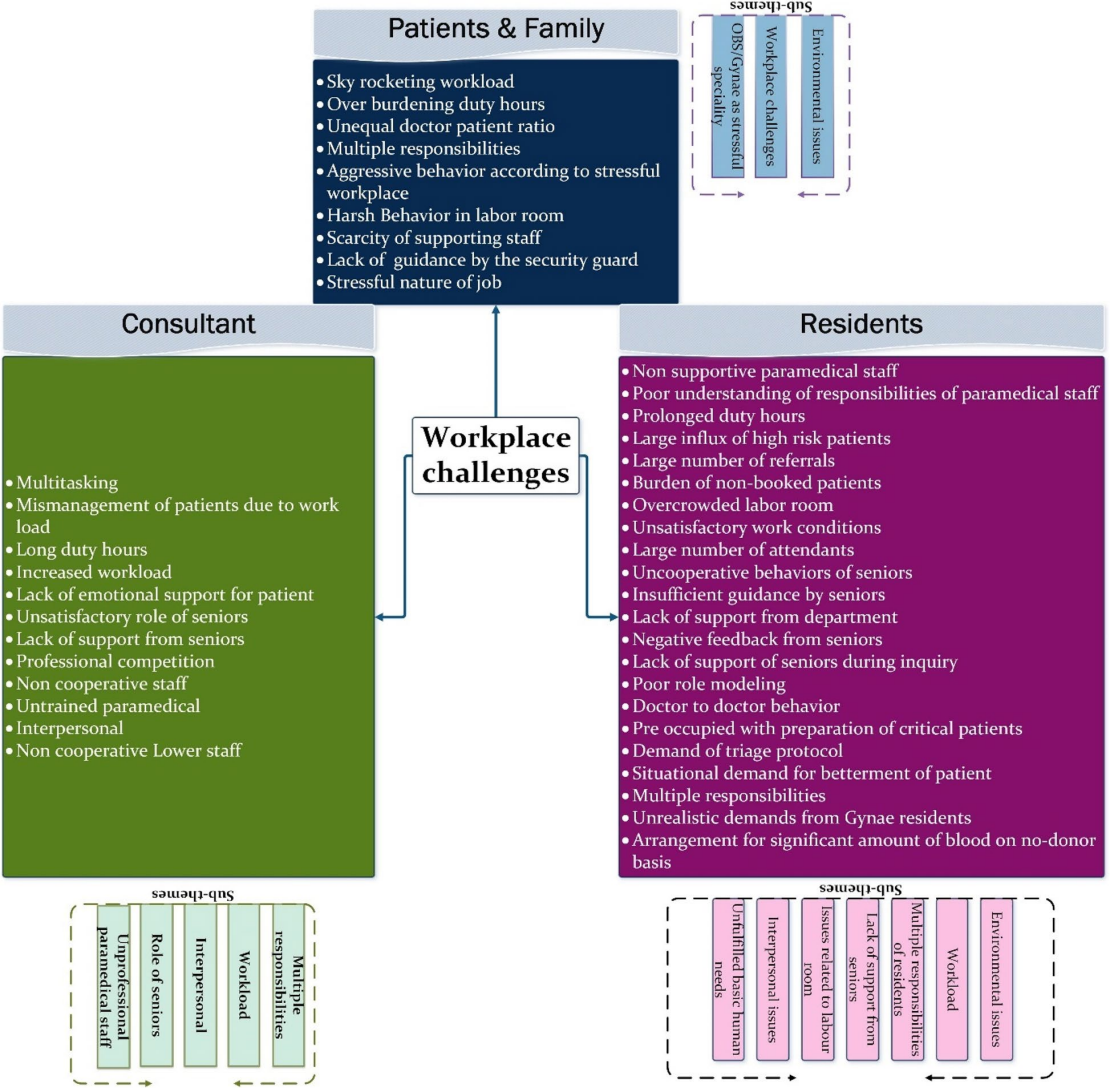
Presenting ‘Workplace challenges’ as a common theme across stakeholders

This theme delves into workplace challenges as perceived by stakeholders, shedding light on potential factors contributing to lapses in professional behaviour, as shown in Fig. 7. Sub-themes include interpersonal dynamics, environmental factors, managing multiple responsibilities, workload issues, the role of senior staff, challenges with untrained paramedical personnel, labor room dynamics, basic human needs, and insufficient support from colleagues and superiors.

**Interpersonal challenges** are frequently highlighted by both residents and consultants. Novice residents struggle with managing conflicts with patients, leading to a tense and stressful environment for all parties involved. This situation is compounded when there is inadequate support from fellow doctors and paramedical staff members.

[Consultant] “Residents and nursing staff did not have good interpersonal relationships and if residents not have nice behaviour with nursing staff they will not get that support from them “ID_6.

Furthermore, the absence of respectful collaboration from other related departments exacerbates the stress and disappointment experienced by residents, ultimately leading to personal frustration that can influence behavioural lapses.

[Resident] In Gynae department our personalities get suppress that even other departments do not respect us. ID_09

Consultants have stressed the importance of training for paramedical staff to offer timely and supportive assistance. They have highlighted the need for paramedical staff to be attentive and responsive, especially during critical emergency situations. Consultants noted that doctors often have to repeatedly request assistance from paramedical staff and may need to raise their voices to convey the urgency of following instructions during critical situations.

[C] “If your paramedical staff is not cooperative then obviously you will get stressed (the doctor not only has to deliver the baby but also send the attendants outside and shout for aya to help her) ID_4.

The physical environment and its related factors significantly impact the satisfaction levels of staff members. Analysis of the data has identified the **"environmental** **factor"** as a common theme from both resident and patient/family interviews, as depicted in Fig. 7. The findings indicate that patients and their attendants recognize the workload of doctors and acknowledge that excessive burden is a primary cause of stress for residents, ultimately affecting their behaviour and well-being.

[P] Duty hours should be reduced and they should get some time for rest and food. There is lot of pressure on them. They start their round at 9am and finish at 2pm because there are 3, 3 patients on each bed. ID_5.

[P] If there are 2 patients on one bed than this will increase anxiety of doc as well. Too many patients are talking simultaneously so this will make her angry. ID_07

[P] On operation day doc is more stressed because she has to manage all the patients and she is alone and feels pressured.” ID_02

The patients have also expressed concern that working hours should be reduced, or the management should consider increasing human resources to cope with the workload. This highlights the interconnection of this theme with other relevant themes.

[P] We should increase the number of doc. The doctor only gets angry when she gets tired and loses her patience level.” ID_07

[P] Patients are coming to doc after 2pm and then they expect their doc to make file but they don’t understand that doc have many other responsibilities.” ID_02

Similarly, from the residents' perspective, it is evident that they are struggling with their workload, and inappropriate physical surroundings further exacerbate their frustrations. Additionally, residents have expressed concerns about issues related to management and counseling of attendants, which adds to their responsibilities. This aligns with the theme discussing various characteristics of attendants, considered significant in creating a conflicting environment.

[R] Unsatisfactory work conditions and patient doctor ratio are among few highlighted by the residents. This can greatly influence the working conditions in support of appropriate environmental factors. A large number of attendants can cause additional management and communication burden for residents to deal with. ID_6.

[R] “Every patient is accompanied by 2 to 3 attendants and you are dealing with patient so every attendant individually comes to us and ask you about the information”. ID_09

**Workload** emerged as a prevalent theme in both residents' and consultants' data as shown in Fig. 7. It is consistently highlighted as a significant issue by both groups in their interviews. Patients and their attendants also expressed concerns about the high workload on doctors. Excessive workload is considered one of the most influential factors contributing to observed lapses in professional behaviour.

[R] “doctors get exhaust because of long 36 h duty, at times we did not eat anything or we could not have proper sleep” ID_01

[R] We are not allowed to refuse any referral even if we do they come back again with the reference of DMS. ID_02 "The biggest problem in this is that we are working in the government sector and there is a human capacity of a doctor, if a doctor has a capacity to check of 5 to 6 patients, but in the government sector, a doctor has to check 20 patients. So the doctor feels overburdened." ID_03.

[R] We have one doctor who is deal 130 to 140 patients from 8 to 2 AM. ID_09 “in other countries they have better working conditions and increase number of doctors because of that they have less work load” ID_09.

The findings discussed above highlight a strong association between the environmental factor and well-being, as well as unmet human needs.

There are **labor room-related issues** highlighted by residents, which can escalate misunderstandings for patients' attendants. Cultural norms often dictate that only female family members accompany the patient during the delivery process. Men are typically not permitted to enter the labor room, especially when female attendants are present or when maintaining the privacy of other patients is a concern. However, this practice can be perceived negatively by patients' male attendants, although it may not reflect the true intent in all cases.

[R] Patients want to have their husbands with them in labour room and in our culture it is not possible. ID_05

[R] “Not allowing male attendants in labor make them think that their patient is in danger”. ID_07

The two overlapping themes identified from residents and consultant data are **'Lack of support from seniors' and 'Role of seniors'**, respectively as shown in Fig. 7. Junior residents have raised concerns regarding the lack of support from senior members. Similarly, consultants have echoed these sentiments, suggesting that seniors are not fulfilling their roles in providing adequate support to junior residents.

[R] " Seniors also pressurize us; they say that you are not examining patients properly or too slowly, because of their responsibility of finishing OPD in given time”. ID_06

Both residents and consultants have emphasized that residents are burdened with **multiple tasks**, some of which consume significant additional time as shown in Fig. 7. For instance, residents often find themselves tasked with arranging blood for seriously ill patients by making excessive calls to relevant blood banks and maintaining patient records. Consequently, they struggle to allocate sufficient time to meet every patient's expectations.

[R] “Residents are always concerned about the patients and they tried to arrange things as soon as they can”. ID_09

[C] “doctor has to be a multitasker, for example if we are short on medicine or blood we send our PGT(post graduate trainee) to blood bank in the middle of the night”ID_1

The findings indicate a lack of support from management in sharing additional responsibilities, while simultaneously expecting doctors to **multitask**. This theme is closely linked to the **'workload' and 'Demand of Gynae specialty'**, contributing to a stressful and demanding environment for residents. Another recurring theme identified from resident and consultant data is the **'Unfulfilled basic human needs'**. Patients and their attendants have also noted the exhausting routines of residents, who often lack time for relaxation or sleep.

[P] “They are human and they are working a lot so they can get tired by working so much” ID_9

[P] “These doctors are so busy with patients that they don’t have time for self” ID_4

Residents have highlighted long duty hours as a significant factor, leaving them with insufficient time and energy to spend quality time with their families. These extended shifts often involve an excessive workload of patients, leading residents to compromise even on their meal times.

[R] “Because of long duty hours and huge number of patients we do not have time to get proper sleep” ID_01

[R] As a Gynae resident we cannot spend proper time with our family we have 30 to 32 h so whatever time we have in our house we have to sleep at that time. ID_09

#### Theme 3: Stressful specialty

**Fig. 8:**
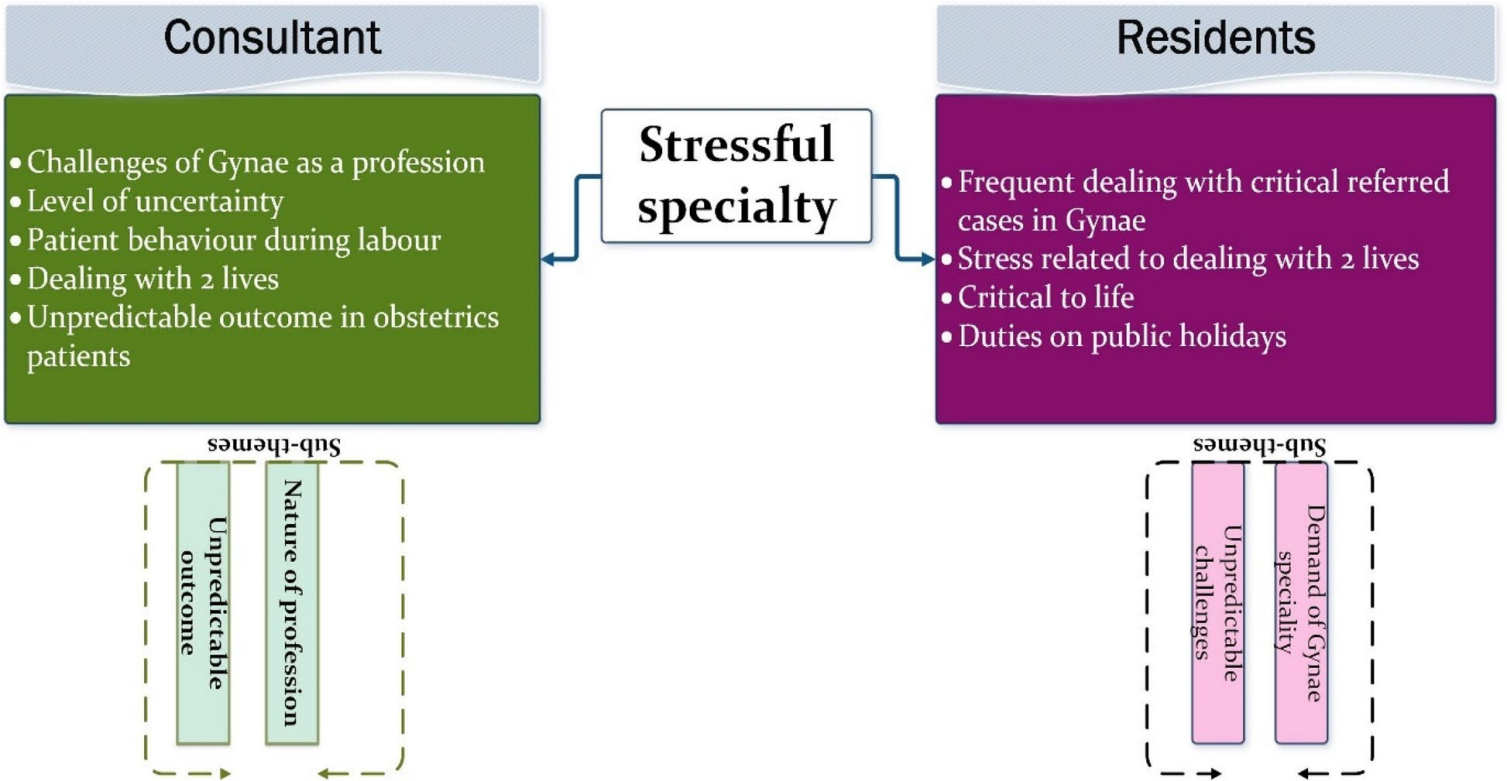
Presenting ‘Stressful specialty’ as a common theme from two stakeholders

This theme, prominently observed in the data collected from both residents and consultants as depicted in Fig. 8, encompasses the sub-themes of 'demand of Gynae specialty', 'nature of profession', and 'unpredictable outcomes related to this profession'. Notably, patients' data only minimally reflects this theme, with only one in 11 patients commenting on the stressful nature of the profession.

Both consultants and residents commonly perceive Gynae as the most stressful domain within the healthcare system, largely due to the simultaneous involvement in the lives of more than one individual. Consequently, this stress may manifest in various ways, potentially leading to lapses in professional behaviour or misinterpretations thereof. The data highlights several specific reasons inherent to the Gynae field, contributing to an overwhelming environment for residents, which can at times be challenging to navigate smoothly.

The unpredictable outcomes associated with Gynae further compound its demanding and challenging nature for those serving within this discipline.

[C] “We have to take care of two lives which is an additional stress for us “ID_6

[R] Residents have high level of tension when patients are non-cooperative, because we have to bear stress of both mother and fetus. ID_07

The interviewed residents have expressed the considerable stress they experience while working in a Gynae department with limited facilities. They highlighted the extra efforts required to arrange supporting resources, such as blood for seriously ill patients. Additionally, residents face the added stress of justifying their efforts and treatments in the unfortunate event of a patient casualty.

[R] “We are aware of our facilities and we have this pressure that even if we examine these excessive number of patients it would be difficult for us to manage them with compromised services, and later we have to justify if the patient expires”. ID_02.

[R] “In our department, our senior PGT herself rushes for the blood of serious patients”. ID_09

The field of Gynae presents unique challenges that are distinct from other areas of the healthcare system. The labor room environment, in particular, poses significant challenges for residents, especially when decisions and treatments are critical to more than one life. Dealing with patients with diverse needs requires patience and experience, as their behaviour can vary greatly during labor, depending on factors such as physical capability, pain tolerance, and cooperation.

Unfavorable workplace conditions can exacerbate the already demanding nature of the work, increasing the likelihood of professional lapses. However, organizational and management interventions can help mitigate these challenges. Improving workplace conditions, adjusting working hours, prioritizing resident well-being, and providing appropriate training are measures that can assist residents in handling such situations more effectively.

Multitasking, another significant challenge, should be minimized by involving support staff and experts at the right time. Residents should not bear sole responsibility for negative outcomes; trained staff should be available to assist them through challenging situations.

Overall, the discussion highlights the interconnectedness of various themes and sub-themes, highlighting the importance of organizational and management decisions in addressing workplace challenges and promoting resident well-being to reduce the occurrence of professional lapses.

#### Theme 4: Cultural

**Fig. 9:**
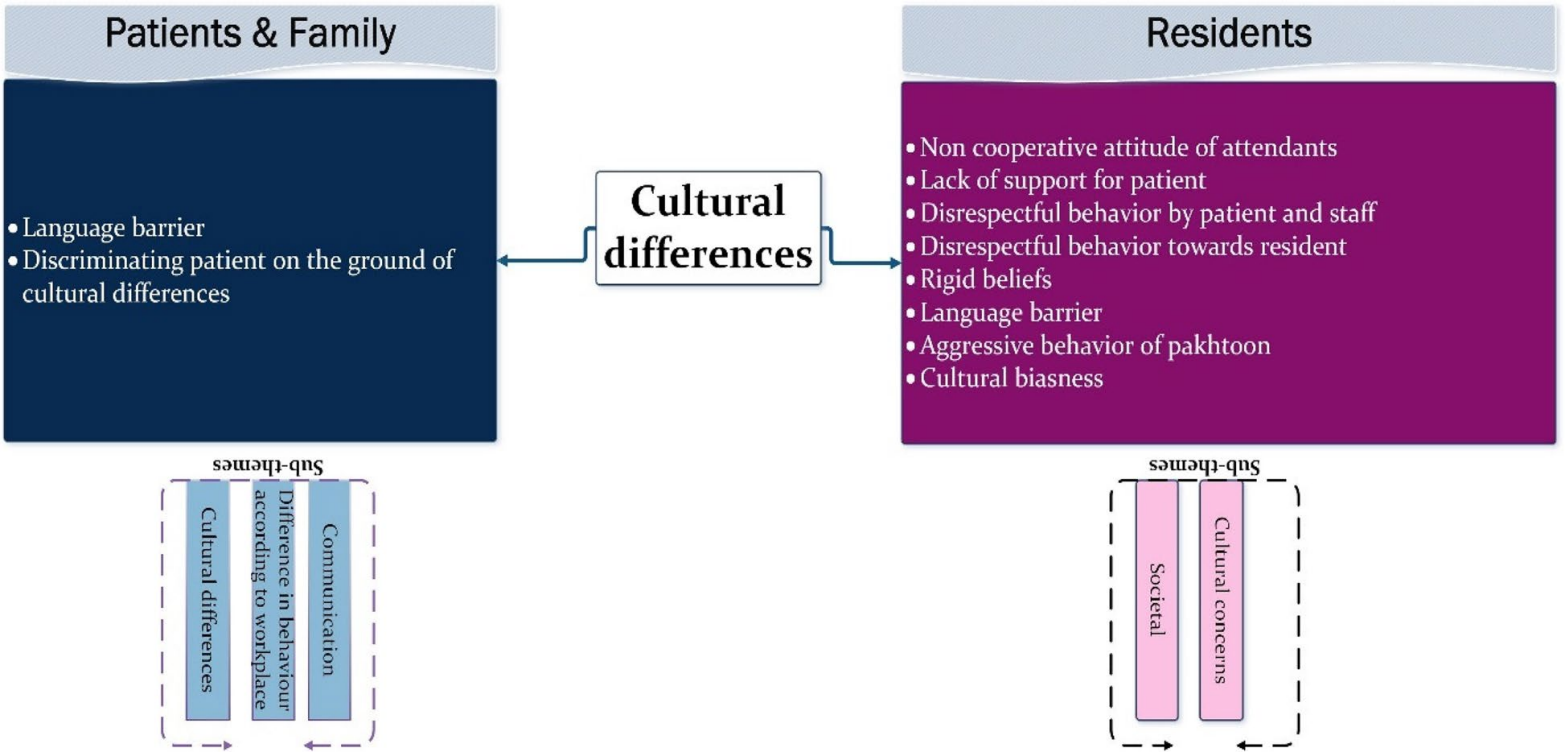
Presenting sub-themes for ‘Cultural differences’ as a common theme across stakeholders’ data

This theme is consistently identified across all three stakeholders, although residents and patients & families place greater emphasis on associated concerns, as depicted in Fig. 9. Notably, the top concerns contributing to lapses in professional behaviour and poor understanding of cultural concerns and communication are highlighted.

Cultural differences can create a discriminatory environment across various aspects, while a lack of understanding of diverse needs can lead to significant misunderstandings between patients and residents. Language barriers, in particular, emerge as a critical cultural factor identified by all stakeholders. Communicating with individuals with different language needs, especially when they adhere to distinctive cultural norms, poses considerable challenges. Consultants also raised this issue during their interviews, although it is merged with 'Patients' characteristics' as a matter of importance.

[C] “Language barrier is a big problem. We also have Pathan (Pakhtoon) population who do not understand”ID-1

[R] “Most of the time Pakhtoon community is very difficult to deal. They do not take your opinion even if they are high risk patients, they force for normal delivery”ID_02

[P] “Doctor in labour room are harsh. they move Pathan attendant out of the labour room and say that we will call you when needed but they don’t call anyone anytime” ID_08

In developed countries, the healthcare system is well-equipped with chaperones who assist healthcare professionals in communicating important information to patients and their attendants. However, in Pakistan, such a system is not in place, making it difficult to expect residents to understand a variety of regional languages. This lack of linguistic diversity among residents can exacerbate feelings of being 'left out' among many patients and their attendants, sometimes leading to arguments with residents.

[R] “We have language barrier with Pakhtoon patients and they are mostly aggressive and thought that we do not understand their issues”. 1D_01

[P] “Nobody allow us in ward if we have to discuss something with doc. Our patient is not educated; she can’t explain her concern in Urdu so how will the treatment proceed.” ID_4

[R] “Most of the Pakhtoon community thought that we are biased and most of the time when they are going back they often say that “we are Pathan that’s why you are not paying attention to us” ID_07.

Furthermore, the language barrier or poor understanding of cultural and regional norms can create a biased environment or negatively influence the thoughts of minorities. This theme is reasonably associated with residents' characteristics and management factors. We believe that the administration should carefully address these important issues and consider providing chaperones to assist residents and patients.

Another challenge for residents arises when dealing with patients whose rigid cultural beliefs influence their mindset. Sometimes, residents must follow recommended treatments to save patients' lives, but attendants may challenge these treatments, leading to conflicting arguments.

Residents also encounter situations where they must take actions to ensure privacy for other patients, especially when multiple patients share a room. However, these actions can be misinterpreted and biased against certain communities. While this issue is partly related to patients' and families' understanding of privacy concerns, it is also the responsibility of the administration and supporting staff to educate them about these sensitive aspects. This burden should not solely fall on residents.

#### Theme 5: Patients’ characteristics

**Fig. 10:**
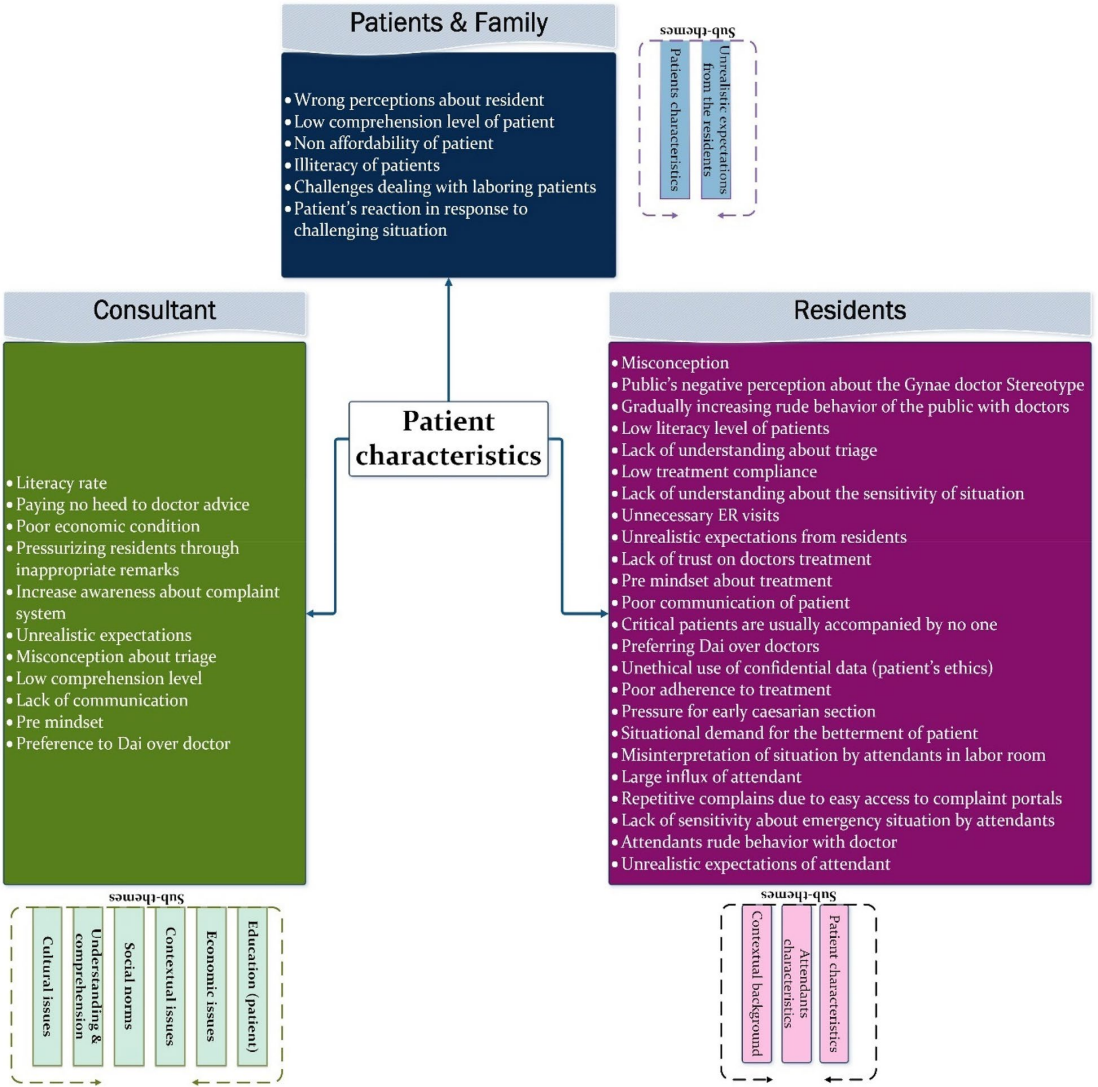
Presenting sub-themes for ‘Patient characteristics’ as a common theme across stakeholders’ data

This theme stands out as one of the most discussed, offering rich information about the specific characteristics of patients and their attendants seeking Gynae treatment, as depicted in Fig. 10. It delves into associated factors attributed to the *educational, economic, and cultural backgrounds *of patients and their attendants, which significantly influence their *behaviour and expectations.* Additionally, it addresses contextual causes that may lead to lapses in professional behaviour.

Pakistan, a developing country, with a literacy rate of 50% and a current birth rate of 3.6 per patient according to PDH 2018 survey, it is a common cultural practice to send senior female members to assist pregnant mothers. This practice is rooted in the belief that experienced females can provide valuable support to new mothers, which is often true. However, it's important not to overlook the fact that patients' attendants are typically the ones who maintain constant contact with residents and serve as the primary point of contact for treatment and critical decisions.

In order to ensure smooth provision of care, it is essential for attendants to cooperate with residents and be capable of interpreting important decisions and communicating needs from both ends. Patients and attendants with ***lower educational backgrounds*** often find it challenging to interpret complex information or navigate to the correct physical location when seeking assistance.

[R] “In government sectors literacy rate of patients are low which creates issue” ID_05

Lack of understanding about the sensitivity of the issue and ***unrealistic expectations*** from residents are frequent reasons that initiate arguments from patients and their attendants. For instance, they may argue if a resident is giving attention to a patient they don't consider to be serious. This behaviour is often perceived as neglectful by patients and attendants, though it may not accurately reflect the resident's intentions.

[R] “suppose we have emergency and I am checking that patient first so all other patients will start shouting, they will not understand the emergency, being a doctor we know that we have to see the emergency patients first but every patient thinks that their problem is the biggest one” ID_01.

In some cases, the unprofessional behaviour exhibited by residents may be a response to ***disrespectful acts from patients or attendants***. Residents have reported instances of disrespect from patients and attendants who fail to grasp the sensitivity of certain treatments and the necessary preliminary measures before administering them. Additionally, the time taken for preoperative preparations may be misinterpreted as a 'delay' in treatment by patients and their attendants.

[R] "The patient complains that the doctor told us that we will have an operation, why don't you operate, if something happens to our child, you will be responsible, patient says" main tumain dekh lounga"( I will see you). They threaten us and misbehave with us".ID_06.

Residents frequently encounter unrealistic expectations from patients and their families regarding infrastructure and facilities, despite these not being within the residents' purview. Patients and their families may expect residents to provide beds and medication, leading to disappointment when residents are unable to fulfill these needs. Arguments where residents are unjustly blamed are unlikely to be accepted by residents and can escalate into conflicts. However, residents and supporting staff should undergo training to effectively communicate any limitations to patients in a more acceptable and understandable manner.

[R] “"When a patient enters the hospital. She doesn't get a bed and medicine. So it is no longer the doctor's responsibility, but because the doctor is in front of them, they feel that the doctor is responsible for these things" ID_06.

It is crucial for residents to recognize the various needs and support required by patients and their families, irrespective of their educational or economic backgrounds. This necessitates appropriate assistance provided to residents from various aspects such as staff, administration, and management support.

Trust is a fundamental element of the patient-doctor relationship, yet it is another concern highlighted by interviewed residents. Patients and their attendants may not fully trust the treatment provided by doctors, possibly due to limited exposure to the healthcare system in rural areas, where self-trained people (called Dai) are preferred over experienced doctors.

[R]”Even if doctor is thinking positive about the patient, patients have this trust issue that might be this doctor will not do my operation properly, or she will make my delivery difficult.” ID_07.

[R] “Patients with low literacy rate go to Dai. ID_09 Daiyon ka jo counselling level ha wo itna acha hota ha k patient kharab kr k bhi bhai jdain to attendant kuch bhi nahi kahe gain unki nazar ma Dai is very good”(counseling skills of Dai are so convincing that even if the patient encountered the complication they thought that Dai is competent). ID_09.

Patients seeking care at public hospitals are often **economically deprived** individuals, burdened with family responsibilities. Many rely on daily earnings, and prolonged absence from work can lead to financial strain. Additionally, they may have caregiving responsibilities that necessitate a swift return home, prompting them to insist on expedited treatment. While these unavoidable circumstances add stress to the situation, appropriate counseling can help patients understand the importance of receiving necessary treatment and alleviate the impact of missing appointments or procedures.

[R] “Patients in Gynae wards are in hurry because they have their family responsibilities and they have small kids at home” ID_07

Both residents and consultants have shared their experiences of navigating **conflicting environments**, which can sometimes lead residents to adopt a harsh tone when communicating with patients and attendants. For instance, in urgent situations requiring aggressive treatment critical to a patient's life, sudden behavioural changes may occur to emphasize the importance of the treatment. This behaviour has a strong link to the attendants' level of understanding and literacy regarding the healthcare system, particularly in the context of Gynae treatment where 2 or more lives may be at risk simultaneously.

[R] “Residents become harsh when attendants or patients do not understand the situation, for example they do not arrange the blood so we have to tell them harshly about it” ID_02

Some senior consultants have provided visionary statements, noting a cultural shift in how the public interacts with medical practitioners. Consequently, there has been a recent increase in aggressive behaviour observed from patients and attendants.

[C] “In past patients and attendants were not so violent but now a days they are violent “ID_6

**Privacy** is a fundamental concern for individuals worldwide, and developed countries have stringent policies and regulations in place to safeguard individual rights. Any breach of these rights can understandably trigger anxiety and concern. Therefore, it is crucial to communicate with patients and attendants about actions that may lead to security breaches involving residents, in order to prevent unpleasant situations.

[C] “Now a days people used to make our videos “ID_6 “people were aware in past as well but now they have got easy access to the portal for complaints”ID_6

This theme is closely linked with the understanding of patients and attendants regarding the healthcare environment, rules, regulations, and treatment specifics that can potentially trigger professional behavioural issues. Management or administration can play a crucial role in providing upfront information to patients and attendants regarding important regulations related to the Gynae specialty.

#### Theme 6: Organisational & administrative

Organizational and administrative authorities are integral in developing effective policies to ensure the smooth operation of healthcare systems. In the context of this study, we believe that the hospital administration can significantly contribute to mitigating the issues discussed thus far. They can take the lead in informing policymakers to design policies relevant to the sensitivity of this field.

**Fig. 11:**
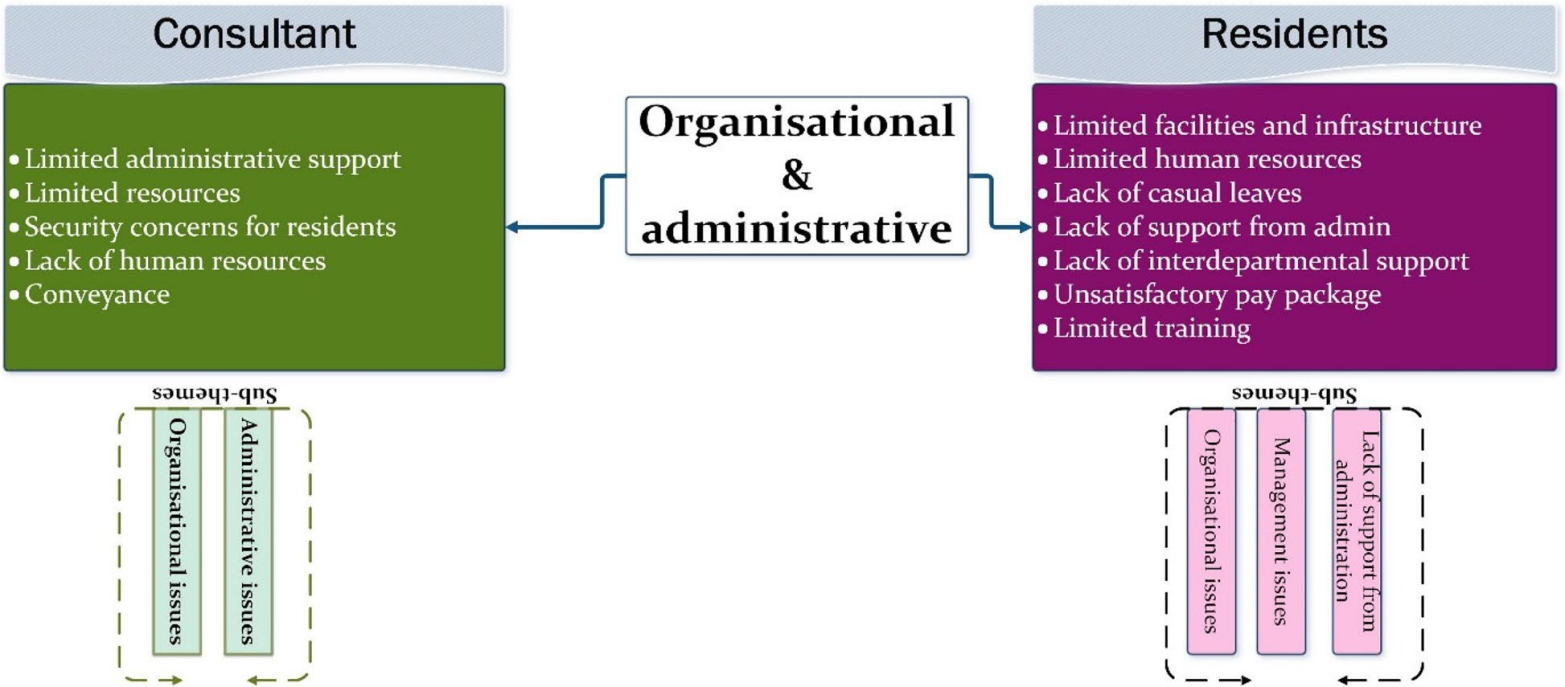
Presenting sub-themes for ‘Organisational& administrative’ as a common theme across stakeholders’data

This theme presents the perspectives of both residents and consultants on organizational and administrative issues that can lead to conflicts and lapses in professional behaviour, as depicted in Fig. 11. Sub-themes include the lack of support from administration and management issues.

An important point to note is the understanding and expectations of patients regarding the provision of facilities and resources necessary for their treatment. During interviews, all patients and families expressed a lack of clarity regarding the roles of hospital administration and doctors.

Both consultants and residents have highlighted challenges including **lack of support from administration, limited resources, and excessive workload**. A high influx of patients can significantly impact working conditions and hinder the ability to provide maximum satisfaction to all patients.

[C] “We do not have anybody who controls the influx of patients outside the rooms or OPD “ID_6

[C] “Also there is the factor that the workload is very high, already you have many patients and doctors are less, sometimes the government does not give you enough resources to facilitate the patients.”ID_3.

[R] “We have two operation theaters and over there we have one anesthesiologist” ID_02

**Administrative support** is crucial in ensuring a secure and pleasant working environment for residents. One concern raised by consultants is the management of patient flow within the hospital. A system should be implemented to queue patients and prioritize them based on their order of arrival for check-ups.

[C] “doctor should be given security, they should be given support, one by one the patient should be sent to the doctor so that the resident also gets time to see the patient”ID_3

The doctor-to-patient ratio varies as per the workload and patient intake. A heavy workload can significantly impact the quality of patient care provided. Most importantly, it can adversely affect the well-being of residents, who are already vulnerable due to the high number of patients they must manage.

[R] “we have huge number of patients and ratio of doctors are much less” ID_01

[R] “we only have one guard for security and we have huge number of patients and every patient have 2 attendants along with her so there is almost 150 people in one room so obviously I will get aggressive”. ID_01.

All of the issues discussed above are likely to influence the professional behaviour of residents making them susceptible to react aggressively which is not a pleasant experience for patients and their family.

## Discussion

The findings from interviews involving three main stakeholders, shed light on potential triggers for unprofessional behaviour discussed in previous sections. The synthesis in the discussion session highlights the key insights gleaned from the interviews, emphasizing the importance of understanding and addressing the challenges faced by residents in their professional journey.

**“Being a resident is not a crime” (ID_C7).**

**“Residency is just like War in battlefield” (ID_C7).**

The quotes from the interviewed consultant underscore the struggles and efforts inherent in the professional life of residents, emphasizing the pivotal role of residency as a preparation for frontline healthcare responsibilities. As residents navigate hierarchical structures within hospitals, they confront diverse challenges and ethical dilemmas, often without adequate training or support. These circumstances can negatively impact their professional behaviour, contributing to a conflicting healthcare environment. It is imperative to comprehensively explore the realities faced by residents to understand and address inappropriate behaviour effectively.

### Demographic inference

The demographic observations from the interviewed stakeholders provide valuable insights into personal issues and patient characteristics that may influence lapses in professional behaviour. For instance, all interviewed patients are housewives with a Matriculation education level, and 45% of them reside in a nuclear family system despite low socioeconomic backgrounds, contrary to societal norms. Consultants, despite higher education and financial independence, are bound to stay in joint families, likely due to the need for familial support in caring for their children amidst demanding work hours. Similarly, a majority of residents reside in a nuclear family system, possibly driven by being unmarried or already established in such living arrangements. These findings highlight a preference for joint family living among Gynae residents and consultants, largely driven by the need for support in childcare due to demanding and long work schedules. However, such living arrangements may also introduce additional stressors related to cultural expectations from family members, as discussed under the 'Personal factors' theme, where stakeholders reflected on their experiences and challenges regarding professional responsibilities and expectations from in-laws.

### Role of competency and communication skills

The interview findings regarding personal factors also examined communication skills and patient expectations. Our study revealed that a lack of communication skills when dealing with challenging situations often led to conflicts. Therefore, we recommend enhancing competency in this area to avoid such situations. This finding aligns with the research by Alipour et al., which also underscores the importance of good communication skills in managing challenging patients [35].

Furthermore, our study found that patients expect physicians to prioritize their needs, respond respectfully to their inquiries and concerns, and uphold their dignity, particularly during sensitive procedures such as those in the delivery room. Patients also value empathy towards their health condition. Similar sentiments were echoed in patient complaints (PCPs), where lack of care or attention from doctors was highlighted. These findings are consistent with prior research emphasizing the importance of communication in maintaining good professional behaviour, with patients specifically emphasizing the significance of being listened to attentively and having medical information explained in understandable terms [17].

Indeed, previous studies have highlighted the impact of inappropriate practices on patients' satisfaction. For instance, practices like insufficient preparation before patient encounters and failure to provide accurate and relevant information have been associated with decreased patient satisfaction [36, 37] Additionally, a common complaint in PCP is the lack of an appropriate number of doctors, which is closely linked to these issues. Addressing these concerns is essential for improving patient care and overall satisfaction levels within healthcare settings.

### Contextual understanding and its influence

The study reveals *context-specific challenges* faced by residents, particularly concerning the *involvement of in-laws* during residency, a phenomenon unique to the cultural context of Pakistani society. Another pertinent issue is the scarcity of affordable housemaids or child-minders. Unlike many developed countries where joint family structures are less prevalent and childcare support is provided by organizations and governments, there are limited initiatives in Pakistan to offer such support. Developed countries have recognized the value of professional child-minders and after-school clubs, providing safe and reliable childcare services to working parents. Unfortunately, Pakistan lacks such reliable facilities, leaving working parents, including residents, with limited options for childcare. While there is a common perception that grandparents can provide adequate care, those without such support often experience stress and uncertainty regarding the safety and care of their children. These stressors are unavoidable for residents and can significantly impact their performance and attitude towards professional responsibilities.

Addressing these context-specific challenges is crucial for supporting residents and ensuring their well-being amidst the demands of their professional roles.

The interview data highlights residents' multitude of responsibilities*, contributing to complaints about long working hours and patient burden, particularly unique to our healthcare system.* *Unrealistic demands placed on Gynae residents,* extending beyond medical care, negatively affect their behaviour due to added burdens. *Labour room issues* arise from societal norms, with family members often leading to miscommunication between doctors and husbands outside the room. *Public distrust* in doctors and teaching hospitals, fueled by negative media portrayal, prompts family intervention and threatening behaviour in the labour room, forcing Gynae residents into defensive positions and hindering treatment decisions.

### Workplace challenges and their impact

The second major complaint in the PCP document, lack of facilities, aligns well with interview data, which highlights various workplace challenges as possible causes of lapses in professional behaviour. These challenges encompass interpersonal, environmental, and workload-related factors, as well as issues with labor room conditions and support from seniors and paramedical staff.

Junior residents' difficulties in handling conflicts with patients are highlighted in our study, reflecting findings from another study in a Gyane department in Pakistan, emphasizing the need for training in interpersonal skills [20]. Additionally, our study emphasizes the negative impact of poor working conditions on residents, a finding consistent with previous research linking organizational and environmental factors to professional behaviour [38].

Excessive workload emerges as a significant factor contributing to observed lapses in professional behaviour, supported by complaints of a huge patient burden in the document analysis. Our results resonate with research from Iran, indicating that high workload and time constraints lead to declining empathy and unprofessional behaviour [39]. Moreover, a narrative review on physician behaviour underscores the role of increased workload in affecting doctor-patient communication and fostering aggressive behaviour [23]. Excessive working hours can also lead to unfulfilled basic human needs and burnout among physicians, as highlighted by stakeholders in our interviews.

Our study identifies workplace issues such as lack of staff support, leading to frustration among residents when instructions are not followed, resulting in perceived lapses in professional behaviour. Patient outbursts often stem from staff not meeting requirements, compounded by language barriers and patient illiteracy hindering communication about triage and treatment priorities. Junior residents' concerns about insufficient support from seniors underscore the importance of role modeling in professionalism, as emphasized by previous research highlighting the impact of role modeling on empathy and professional behaviour [38].

### Distinctive stresses associated with this specialty

The study acknowledges existing literature linking high-pressure specialties like surgery and Obs and Gynae with violent behaviours due to unique stresses [14]. Gynae residents, consultants, and even patients and their families described the profession as inherently stressful and unpredictable. This study highlights how professional behaviour is influenced by the stress of managing the lives of both mother and baby. Additionally, factors such as a high influx of non-booked patients, complicated referrals, unrealistic patient expectations, limited resources, non-cooperative attendants, and unmet human needs contribute to the already stressful environment in our healthcare setup.

### Cultural differences and their importance

In developed countries, the healthcare system provides chaperones to assist healthcare experts in communicating vital information to patients and attendants. However, in Pakistan, such a system is absent, making it difficult to expect residents to understand various regional languages like Pashtoo and Gilgiti. This language barrier can exacerbate feelings of exclusion among patients and attendants, leading to conflicts with residents. This finding underscores the complexity of professionalism as a social construct, emphasizing the need to consider context, geographic location, and culture when addressing lapses in professional behaviour [40].

### Why it is important to consider patients characteristics

This study delves into the specific characteristics of patients and attendants, extracted from interviews and unique to this contextual study. Their educational, economic, and cultural backgrounds vary significantly, posing challenges for residents in their interactions. Patients typically have limited education and come from low socioeconomic backgrounds, with predetermined expectations and a sense of entitlement to care due to taxes paid. Accompanied by numerous attendants, they often hold fixed views about doctors' duties. This perception, evident in PCP complaints, contributes to conflicts with doctors, exacerbated by varying tolerance levels. Notably, literature lacks data on patients' perspectives regarding residents' professional behaviour. Remarkably, patients and families do not attribute lapses in professional behaviour to organizational/administrative issues but instead fault residents for perceived deficiencies in hospital facilities and medication availability, likely due to a lack of awareness about administrative roles—a finding specific to impoverished patients in public sector hospitals.

The patient holds a central role in the healthcare system and is a significant stakeholder whose perception of lapses in professional behaviour is explored in this study. This encompasses actions such as imposing additional financial burdens (e.g., recommending costly tests, requiring the purchase of operation accessories) and unnecessarily prolonging hospital stays. Particularly poignant is a patient's observation regarding class-based social discrimination among patients, where "the rich were preferred over the poor," highlighting disparities in access to facilities and guidance for attendants.

### Role of organisational & administrative support

Organizational and administrative authorities are pivotal in providing effective hospital facilities, a theme emphasized by residents and consultants in this study. Issues such as limited infrastructure, uncooperative senior behaviour, absence of casual leaves, and inadequate salary packages relative to workload are identified as factors negatively influencing professional behaviour. This underscores the insufficient support provided by the administration, training systems, and external factors, which are perceived as more influential than personal factors. These findings align with other studies that highlight deficiencies in training hospital systems as contributing to lapses in professional conduct among residents [41]. Additionally, organizational deficits, such as inadequate facilities and poor security, along with a gap between administration and clinical departments, are identified as sources of conflicts [20]. Overall, these results support the notion that professionalism is situation-sensitive, and the training environment may contribute to the deprofessionalization of residents, as observed in literature [42–44].

#### Uniqueness and importance of this study

To the best of our knowledge, this study represents a pioneering effort in thoroughly evaluating the perspectives of patients, consultants, and residents regarding lapses in professional behaviour among Gynae residents in teaching hospitals in Pakistan. Existing literature underscores the significance of complaints in uncovering underlying issues and guiding solutions. Studies by Rogers et al. and Hoffmann et al. have highlighted the prevalence of complaints related to medical professional behaviour in various contexts [45]. Our research complements these studies by providing deeper insights from multiple perspectives. While professionalism is influenced by environmental and personal factors, literature lacks sufficient data on patients' expectations of doctors. Our study fills this gap by exploring Pakistani patients' perspectives on the factors contributing to lapses in professional behaviour among residents, thereby enriching understanding in this area. Additionally, our study integrates document analysis with interview data to provide a comprehensive analysis.

#### Contribution of the study

The paradigm shift in understanding professionalism lapses highlights the increasing importance of incorporating patients' perspectives. Our research offers valuable insights from key stakeholders, which can serve as a benchmark for addressing identified issues in healthcare. It emphasizes the need for training residents in professionalism, with educators leveraging the findings of patient complaints to inform their approach.

Traditionally, resident misbehaviour has been attributed to character flaws [46, 47] but our study reframes this interpretation within the context of training systems and environments. This nuanced approach enables a deeper understanding of the phenomenon and opens avenues for incident prevention through educational and organizational interventions. While some findings may not be universally applicable, our approach to addressing trainee professional lapses holds implications for diverse educational settings.

## Conclusion

The findings of our study disclose several perceptions as a possible influential cause of unprofessional behaviour of residents. Excessive workload, workplace challenges is the residents' most frequently mentioned contributing factors. Another interesting finding of this research is an emerging theme related to the characteristics of patients and attendants which has been attributed by both consultant and resident. These characteristics can be considered useful in understand the causes and implications of conflicting environment. Opinion from administration or higher authorities regarding issues faced by residents and patients offer insight for future work.

### Implication for educational practice

This section highlights key lessons learned from the comprehensive interview datasets involving three stakeholders, accompanied by recommendations derived from the research.

- Balancing professional commitments with family responsibilities is crucial, as it varies among individuals and can significantly impact professional behaviour.
- Management's attention to residents' well-being is paramount, as an unsupportive working environment can negatively affect professional conduct.
- Implementing a mentoring scheme for junior members, allowing them to shadow seniors, can enhance their ability to handle complex situations effectively.
- Providing trained paramedical staff to assist residents can alleviate their workload, allowing them to focus on critical tasks and improve patient care.
- Ensuring supportive working conditions, including physical environment and workload management, can enhance resident well-being and positively influence professional behaviour.
- Recognizing the unique challenges of the Gynae specialty is essential for developing policies that support stakeholders in this field.
- The specific characteristics of patients and families identified in this study underscore the need for further exploration of their beliefs, understanding, and expectations to address diverse cultural and social needs.
- Addressing the specific needs of all stakeholders, including privacy, language comprehension, socio-economic factors, and personal well-being, can foster a harmonious environment and mitigate conflicts.

### Strengths and methodological challenges

Major limitation rather challenge of my study was the difficulty in understanding the construct by patients and family. Although all are matriculation by education but they unable to comprehend that there are always some factors behind the some behaviour. It might be a reason of less rich data as compared to consultants and residents. Another limitation might be some element of social desirability bias in patients because we collected data from admitted patients. Although researchers were unbiased but there might be some fear that telling the truth might affect their treatment procedures or behaviour of resident in charge of their ward. Only 2 public sector hospitals were targeted which restrict the scope of the study. Moreover, almost all the participants were female except for 1 male resident (subject to availability) pose gender-related limitation.

### Implications for further research

The research on professionalism in healthcare departments in Pakistan has been limited, leaving several aspects unexplored regarding the reasons behind poor behaviour exhibited by both doctors and patients. A multicenter study involving Gynae departments across various public and private sectors in different cities of Pakistan would provide a more comprehensive understanding of the prevalence, risk factors, significance, and consequences of lapses in professional behaviour.

Future research should also consider including perspectives from paramedical staff, hospital administration, and senior consultants within the Gynae unit to gain a holistic view of the issue.

Given the limited presence of male residents in Gynae obstetrics training, reaching out to them for their perspectives and comparing them with female residents could provide valuable insights.

Additionally, further research focused on remedying the issues identified in this study would be beneficial for the medical education community in addressing professional behaviour concerns effectively.

## Data Availability

All datasets used and/or analysed during current study available from corresponding author on reasonable request.
